# Breathable and wearable graphene/waterborne polyurethane coated regenerated polyethylene terephthalate fabrics for motion sensing and thermal therapy

**DOI:** 10.1186/s11671-024-04004-w

**Published:** 2024-04-04

**Authors:** Zhou Zhang, Xuzhen Zhang, Wenjian Huang, Xiong Zheng, Bona Ding, Xiuhua Wang

**Affiliations:** 1 National Engineering Laboratory for Textile Fiber Materials and Processing Technology (Zhejiang), Zhejiang Sci-Tech University, Hangzhou, 310018 People’s Republic of China; 2 Zhejiang Provincial Innovation Center of Advanced Textile Technology, Shaoxing, 312030 People’s Republic of China

**Keywords:** Regenerated PET, Composite fabric, Graphene, Waterborne polyurethane, Coating

## Abstract

The functional utilization of recycled polymers has emerged as a current prominent and timely subject. Flexible wearable devices with high sensitivity to conductivity have garnered significant attention in the fields of human healthcare monitoring and personal heat management. One significant obstacle that needs to be addressed is the simultaneous maintenance of both sensing functionality and durability in composite fabrics. In this paper, a collection of durable, breathable, and flexible smart fabric was produced using the scratch coating method. The fabrics were created by utilizing a regenerated polyethylene terephthalate fabric as a base material, incorporating graphene microsheets (G) as a conductive agent, and applying a waterborne polyurethane layer as a surface protective coating. Furthermore, an investigation was conducted to assess their sensing performance and electrothermal performance. The composite fabric exhibits significant advantages in terms of high conductivity (592 S/m), wide strain range, high sensitivity (Gauge factor = 6.04) and fantabulous dynamic stability (2000 cycles) at a mass ratio of Graphene/WPU loading of 8:2. These sensors were successfully utilized to monitor various degrees of real-time human body movements, ranging from significant deformation bending of elbows to slight deformation swallowing. Furthermore, the sensors also exhibit a significant electric heating effect. Specifically, when a voltage of 10 V is applied, the sensors can reach a steady state temperature of 53.3 °C within a mere 30 s. This discovery holds potential for the development of wearable heaters that can be used for on-demand thermal therapy, functional protective clothing, and medical electric heating wearables.

**Supplementary Information:**

The online version contains supplementary material available at 10.1186/s11671-024-04004-w.

## Introduction

The investigation into regenerated polymers aligns with the internationally advocated principle of promoting environmentally friendly energy conservation [1–3]. Among these, regenerated polyethylene terephthalate (rPET) stands out as the most significant category of regenerated polymers [4]. However, its current applications still primarily remain traditional. The exploration of potential value-added functional utilization for rPET has emerged as a new focal point. With the rapid advancement of wearable electronics, there has been a significant surge in interest in flexible sensors. This is primarily attributed to their extensive potential applications in various fields, such as human healthcare monitoring [5, 6], electronic skin [7, 8], soft robots [9, 10], and personal thermal management [11–13]. As widely acknowledged, flexible sensors are commonly found in various forms such as aerogels [14, 15], hydrogels [16, 17], films [18, 19], and textiles [20, 21]. However, the rapid advancement of flexible wearable sensors has been significantly hindered by various challenges, including low mechanical strength [22], poor breathability, and lack of comfort [23]. These limitations have substantially restricted the application of aerogels, hydrogels, and films in this field. Therefore, seamless integration of wearable fabric sensors with conventional textiles that encompasses aesthetics, comfort, and electronic functionality presents significant potential for flexible sensor applications [24, 25].

To fabricate smart textiles with remarkable flexibility and sensing capabilities, the current methodology revolves around enhancing the conductivity of insulating yarns and incorporating them into a textile framework using weaving, knitting, and sewing techniques [26, 27]. The functional components commonly found in sensors include metallic threads [28], conductive polymers [29], and carbon materials [30]. Graphene, known for its exceptional electrical conductivity, thermal conductivity, and electrothermal effect [31], possesses extensive utility in the realm of sensors and Joule heating devices. Khadka et al. [32] developed wearable heaters by utilizing Han paper coated with graphene nanosheets due to the high conductivity and rapid heating properties of graphene, which make it suitable for providing heat supply in human-body thermotherapy. Liu et al. [33] tailored the performance of self-regulating heating devices by incorporating a secondary thermoplastic elastomeric phase into polymer composites filled with graphene nanoplatelets. The objective of their research was to optimize the flexibility and self-regulating heating functions of these devices. However, the practical implementation of graphene is significantly challenged by its propensity for discontinuous distribution. To address the issue of discontinuity, it is widely adopted to utilize graphene in suspension form to coat the substrate, thereby forming a continuous conductive microlayer [34]. Despite its weak adhesion to polymer substrates, peeling often occurs during application procedures.

Liu et al. [35] utilized a direct laser writing technique to directly deposit graphene onto polyimide fabric for the fabrication of a strain sensor. However, this approach was found to have a significant drawback, namely the weak bonding between graphene and fabric. Singh et al. [36] investigated the chemical bonding and electronic network of reduced graphene oxide when reacting with compounds that possess active functional groups, with the aim of securely anchoring it onto the surface of fabric. A double-layer mesh microstructure was additionally devised to effectively achieve the multi-point measurement of micro-pressure, thereby enhancing electrical conductivity while maintaining softness [37]. Nevertheless, the complexity and limited practicality present challenges for achieving widespread adoption; water-based polyurethane has emerged as a promising interface adhesive to address these aforementioned concerns due to its cost-effectiveness and high adhesion [38].

In this work, a scratch coated fabric was synthesized by integrating with both sensing capabilities and joule heating functions. By optimizing the fabrication technique and finding the optimum ratio of graphene and waterborne polyurethane mixing-coating, this work aimed to investigate the properties of flexible sensing and thermal response properties composite knitted fabrics. The utilization of graphene nanosheets, which possessed excellent electrical conductivity and flexibility, enabled the achievement of high electro-thermal efficiency. Furthermore, the incorporation of waterborne polyurethane maximized the binding property, thereby enhancing the abrasive resistance of the composite fabric. In the mixed slurry system, graphene lamellar structure acted as rigid nodes, while the WPU acted as soft chain segments. The synergistic combination of these two components resulted in the formation of a robust conductive network structure, thereby facilitating the strong bonding of the conductive composite fabric.

## Experimental section

### Materials

Waste PET fiber and scrap were provided from Zhejiang Guxiandao Polyester Dope Dyed Yarn Co., Ltd, China. Waste PET were processed using an integrated batch polycondensation reactor combining glycolysis in ethylene glycol with catalysts, and then polycondensation with stabilizers, resulting in spinnable rPET [39]. After preparing the stretch yarn through a horizontal micro-extrusion machine and drafting device, the regenerated PET knitted fabrics (rPET, 120 g/m^2^) were woven using a hosiery knitting machine. Graphene slurry (diameter 5 − 20 μm, layer thickness 2–5 nm, purity 99.5%, weight ratio 10 wt %) was purchased from Nanjing XFNANO Materials Tech Co., Ltd., Nanjing, China. WPU with 40 wt% was purchased from Shanghai Macklin Biochemical Technology Co., Ltd. All materials were used without further purification.

### Preparation of graphene/WPU composite coating slurry

**Fig. 1:**
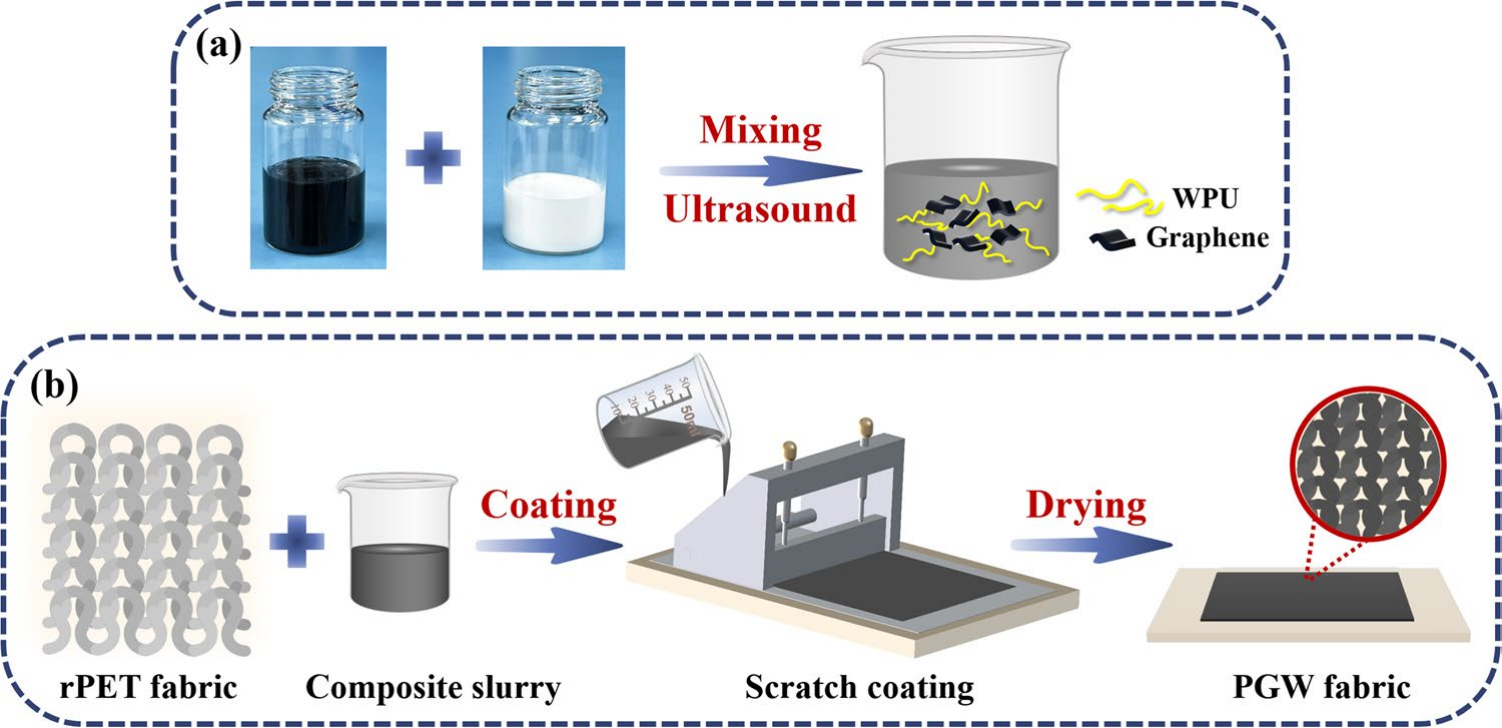
Schematic illustration of preparation process of **a** G/WPU composite slurry and **b** PGW composite fabric

Given amount of WPU dispersion and deionized water were mixed and stirred ultrasonic at room temperature for 2 h. Graphene slurry was fully emulsified using homogenizer, then mixed with deionized water for ultrasonic 2 h to prepare solutions with various concentrations. Subsequently, WPU aqueous solution was prepared with different content of graphene nanosheets as shown in Table 1. Graphene/waterborne polyurethane (G/WPU) suspension was firstly mechanically stirred with a speed of 300 rpm at 20 °C for 1 h, and then ultrasonication treated (600 W, 40 kHz) for 4 h. The diagram of stable composites slurry with various content of G/WPU suspension obtained was presented in Fig. 1 a.

**Table 1:** Preparation parameters of coated composite fabrics

Notation	Mass ratio of G/WPU	coating weight (g/m^2^)
PGW-1	4:6	16.03
PGW-2	5:5	16.77
PGW-3	6:4	15.07
PGW-4	7:3	14.23
PGW-5	8:2	11.97
PGW-6	9:1	11.57
PG	10:0	11.10

### Preparation of graphene/WPU coated rPET fabrics

The specific blade coating process of the G/WPU composite fabric was illustrated in Fig. 1b. Before coating, neat knitted rPET fabrics were soaked in 75% ethanol solution and cleaned in an ultrasonic cleaner at 80 °C, 80 kHz and 500 W for 30 min to remove the oil on the fiber surface, washed thoroughly with deionized water and dried in an oven at 60 °C. The coating speed was set at 90 cm/min at 60 °C to achieve coating samples with a thickness of 0.45 mm. The obtained samples were dried at 120 °C for 30 min, and then repeat the scraping once to obtain the coated rPET fabric.

The assessment of the coating results was based on the parameter known as coating weight* W* (g/m^2^), and which could be calculated as following:

$$W = \frac{{W_{1} - W_{2} }}{A}$$

where *W*_1_ refers to the weight after scratch coating process, *W*_2_ refers to the pristine weight of control sample, and *A* refers to the surface area of the original sample.

### Characterization

*Morphology* Scanning electron microscopy (FE-SEM, JEOLJSM-840, Japan) and stereo microscope were operated with an EHT of 3.0 kV and a work distance of 8.0 mm to observe the surface and cross section morphology of G/WPU and explore the interface combination of components.

*Chemical structure and thermal stability* Chemical structure of rPET fabrics before and after scratch coating was measured through Fourier transform infrared spectroscopy (FTIR, Nicolet 5700, Thermo Electron Corporation, USA). The test wavenumber range of nanometer powder was 400–4000 cm^−1^ with a resolution 0.4 cm^−1^.

The thermogravimetric analyzer (TG209 F1, Netzsch, Germany) was employed for thermogravimetric analysis (TGA) in nitrogen atmosphere with a heating rate of 10 °C/min across a temperature range of 30–800 °C. The thermal stability of composite fabrics was determined by analyzing the TGA and derivative thermogravimetry (DTG) curves.

*Physical properties test* Mechanical measurements of fabrics were tested at room temperature using a fabric strength tester (YG026Q). All samples were placed in a standard environment (temperature 25 °C, moisture 65%) for a duration of 24 h to mitigate the potential influence of moisture on the mechanical properties. The dimensions of fabric sample were cut as 80 mm long, 20 mm wide and 5 mm thick. The constant stretching rate was set as 100 mm/min, and the initial distance between the two clips as 50 mm.

*Water vapor permeability test* In order to assess the breathability of textile sensor, it was placed on top of a beaker filled with hot water, allowing unobstructed passage of water vapor through the fabric. The ASTM E96 inverse cup standard [40] was used to accurately measure its breathability by exploring the water vapor transmittance (WVT) rate. Specifically, the textile sensor was exposed to a temperature of 38 °C, relative humidity of 50%, and wind velocity of 1 m⋅s^−1^. The WVT rate was calculated as following:

$${\text{WVT rate}} = \frac{{m_{1} - m_{2} }}{S} \times 24$$

where *m*_1_ represents the initial weight of beaker, *m*_2_ represents the final weight of beaker, and *S* represents the test area.

*Sensing and electrothermal performance test* The composite fabrics were connected to the circuit to measure the sensing performance via a sourcemeter (Keithley 2450, USA). In addition, the Joule heating behavior of the composite fabrics (dimensions of 2 × 1 cm^2^) were incorporated into a circuit connected with a DC bias power source under 1–20 V for thermotherapy. Unless otherwise stated, each experiment was conducted at an ambient temperature of 25 °C and a humidity of 50%.

## Results and discussion

### Morphology and structural characterization of PGW

**Fig. 2:**
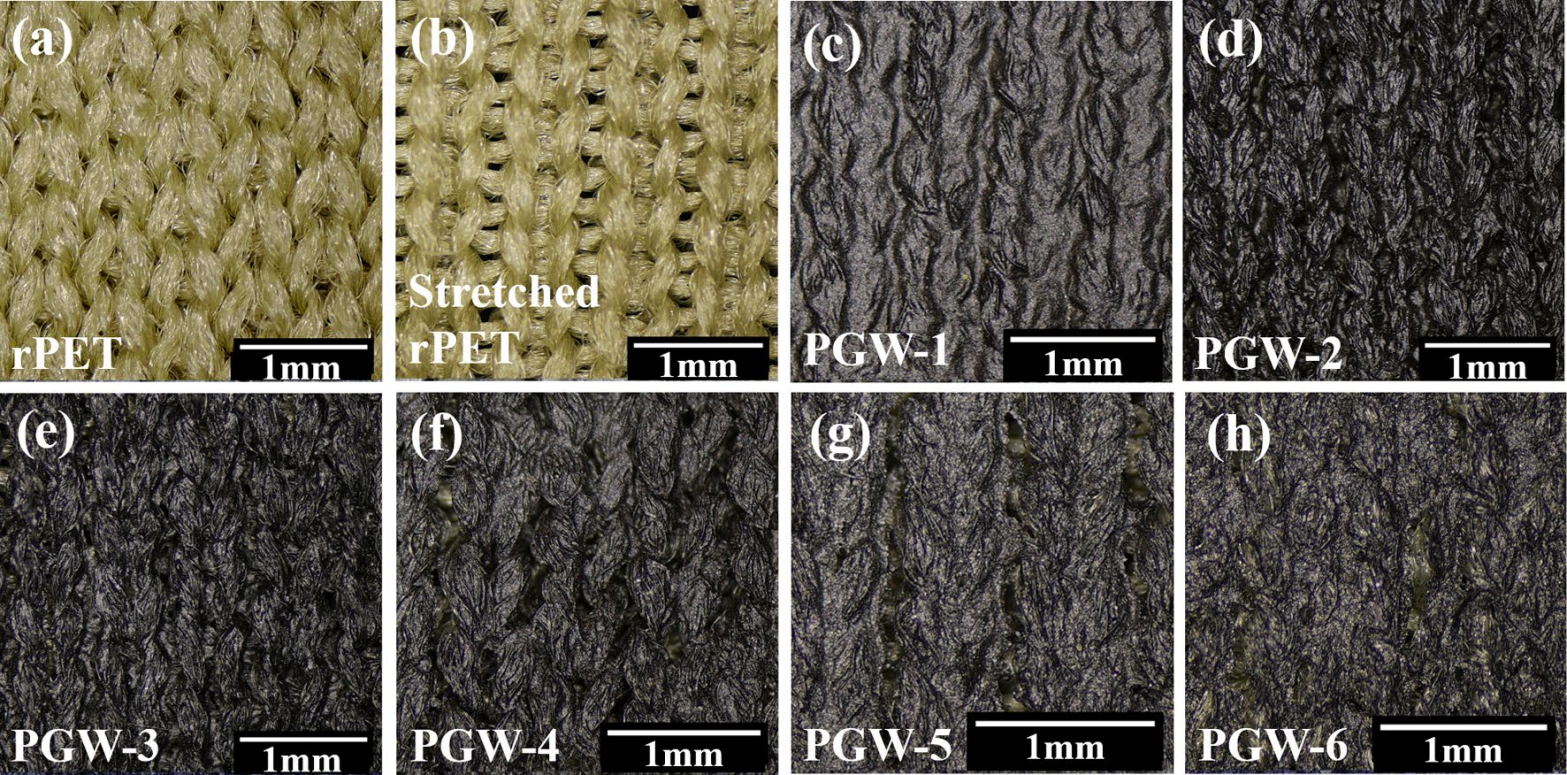
Stereo morphology images of **a** rPET, **b** stretched rPET, **c** PGW-1, **d** PGW-2, **e** surface of PGW-3, **f** PGW-4, **g** PGW-5, and **h** PGW-6

**Fig. 3:**
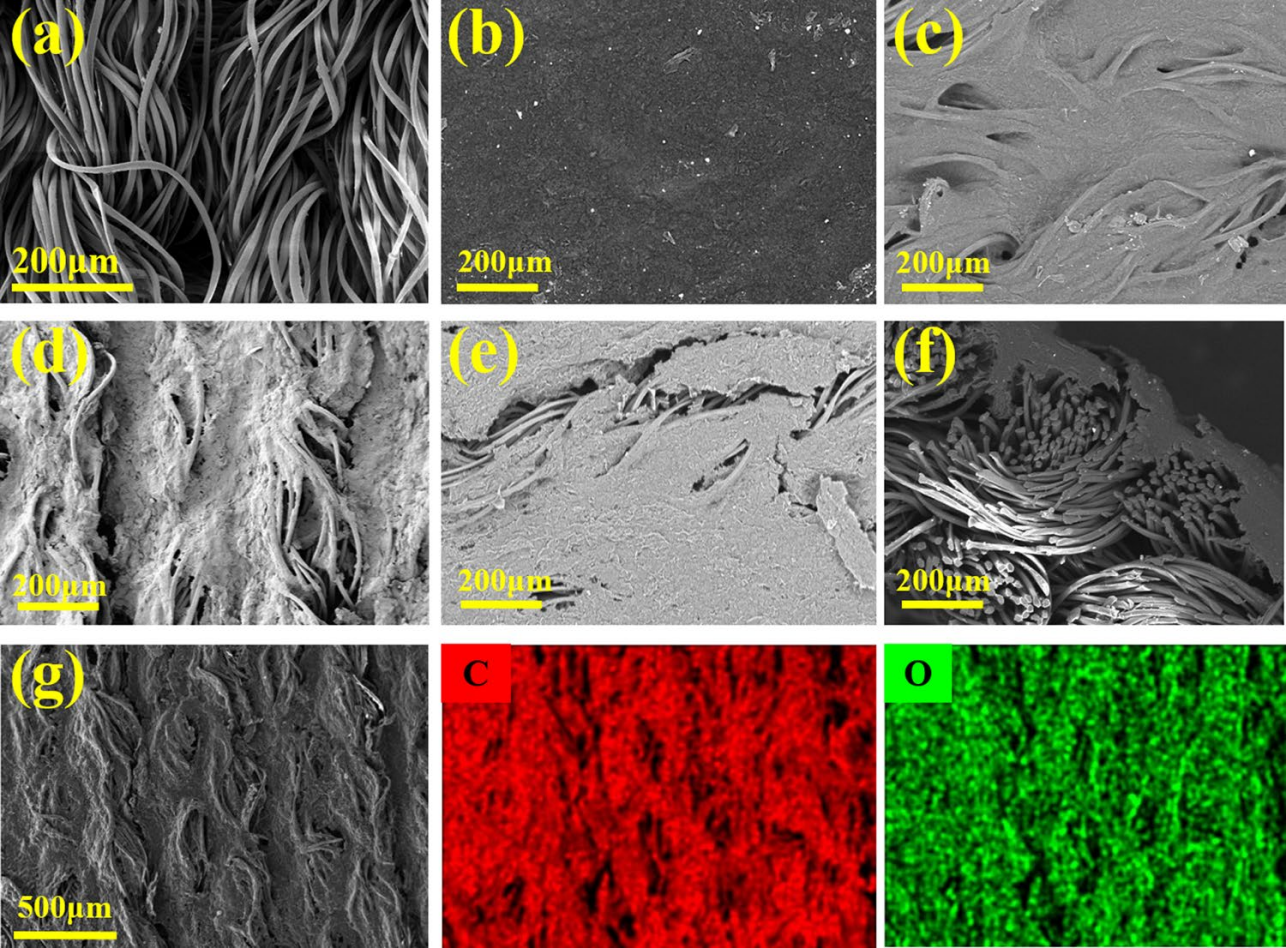
Lateral morphology SEM images of **a** rPET fabric, **b** PGW-3, **c** PGW-4, **d** PGW-5, **e** surface of PGW-6, **f** cross section of PGW-5, and **g** mapping of C and O elements on the surface of PGW-5

The macroscopic and microscopic surface morphology and cross section morphology of rPET, PGW-1, PGW-2, PGW-3, PGW-4, PGW-5 and PGW-6 samples can be observed respectively through stereo microscope images (Fig. 2) and the high-resolution FE-SEM images (Fig. 3). As shown in Fig. 2a, b, lots of fibers in rPET fabric were cross-linked independently. Yarns were bent into loops with a porous structure, thus achieving a good air permeability. In the flexible composite fabric (PGW), rPET fiber acted as the core tightly bonded to the shell G/WPU. In G/WPU composites, WPU acted as an interface adhesive to firmly bond graphene with rPET substrate based on a similar solubility parameters of WPU and rPET [41], forming a strong interaction and playing a "bridge" role between graphene and rPET. In Fig. 2c, due to the strong interaction, WPU was closely attached to rPET, resulting in a dense cross structure with few pores and weakened air permeability of PGW-1 composite fabric. With an increase in the proportion of graphene in G/WPU, PGW-5 composite fabric led to an augmentation in pore size after coating (Fig. 3d), thereby enhanced the air permeability of the flexible composite fabric. The EDS elemental analysis in Fig. 3g revealed that a uniform distribution of C and O elements on the surface of rPET, indicating the uniform load of WPU and graphene nanosheets on its surface and demonstrating the feasibility of preparing composite fabric by scratch coating method. However, as illustrated in Fig. 2h and Fig. 3e, an increase of graphene content in G/WPU resulted in a detachment of the coating from rPET fabric. Fiber traces could be gradually observed with some cracks appeared between the coating and fabric, shown in Fig. 3d and e. Meanwhile, the adhesion of coating to rPET became deteriorate, showing a weakened bonding between WPU and rPET fabric.

### Chemical structure and thermal properties

**Fig. 4:**
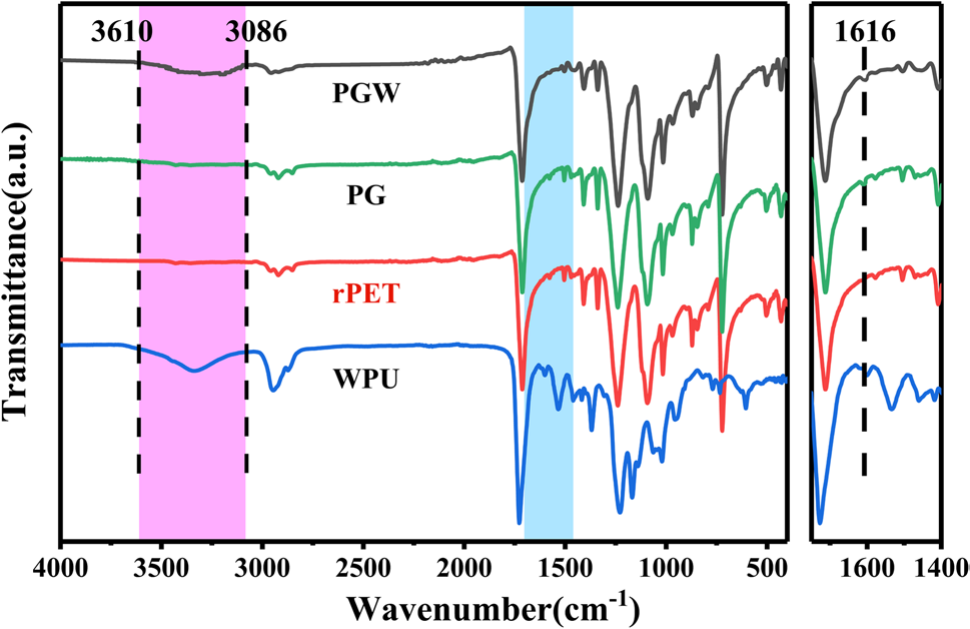
ATR-FTIR spectra of WPU, rPET, PG and PGW

The ATR-FTIR spectra of virgin rPET fabrics and G/WPU coated rPET fabrics were investigated in the range of 400–4000 cm^−1^, as shown in Fig. 4. The characteristic peaks of rPET at 2920, 1730 and 1409 cm^−1^ were attributed to the stretching vibration of methylene (CH_2_), the stretching vibration of carbonyl group and the in-plane bending vibration of benzene ring C–H [42]. In addition of graphene and waterborne polyurethane led to the appearance of several new peaks in the spectra of the composite fabrics. The peaks ranged in 3300–3600 cm^−1^ were related to N–H tensile vibration in polyurethanes and extremely sensitive to hydrogen bond [43, 44]. Among them, the N–H characteristic peak value of binding with hydrogen atoms provided by hydroxyl and carboxyl groups in rPET can be observed at 3330 cm^−1^, while the peak around 3450 cm^−1^ was contributed by free N–H characteristic peak. A broad absorbance peak between 3610 and 3086 cm^−1^ was ascribed to N–H/O–H stretching vibration in Fig. 4 [45]. The FTIR spectra of PG fabrics were much similar to those of rPET fabrics. There was likely no chemical reaction between graphene and rPET, relying mainly on the strong adhesion of WPU to bind graphene firmly to the surface. For the spectra of PGW in Fig. 4, the abroad absorption bands at 3250 cm^−1^ were assigned to –OH stretching vibrations of residual water [46]. Besides that, the absorption peak at 1616 cm^−1^ in the infrared spectrum could be attributed to either the C=C stretching of the benzenoid rings in graphene or in the diisocyanate in WPU. To analyze the presence of graphene, morphology SEM images of the composite fabrics were used in combination with ATR-FTIR spectra. As shown in Fig. 3d and Fig. 3e, the composite fabric surface exhibited uniform distribution of micron-sized particles, with a high content of 80% C element, as indicated by the C element mapping on the surface of PGW-5. The existence of carbonyl group, N–H and C=C characteristic peak demonstrating that PGW composites had been successfully fabricated.

**Fig. 5:**
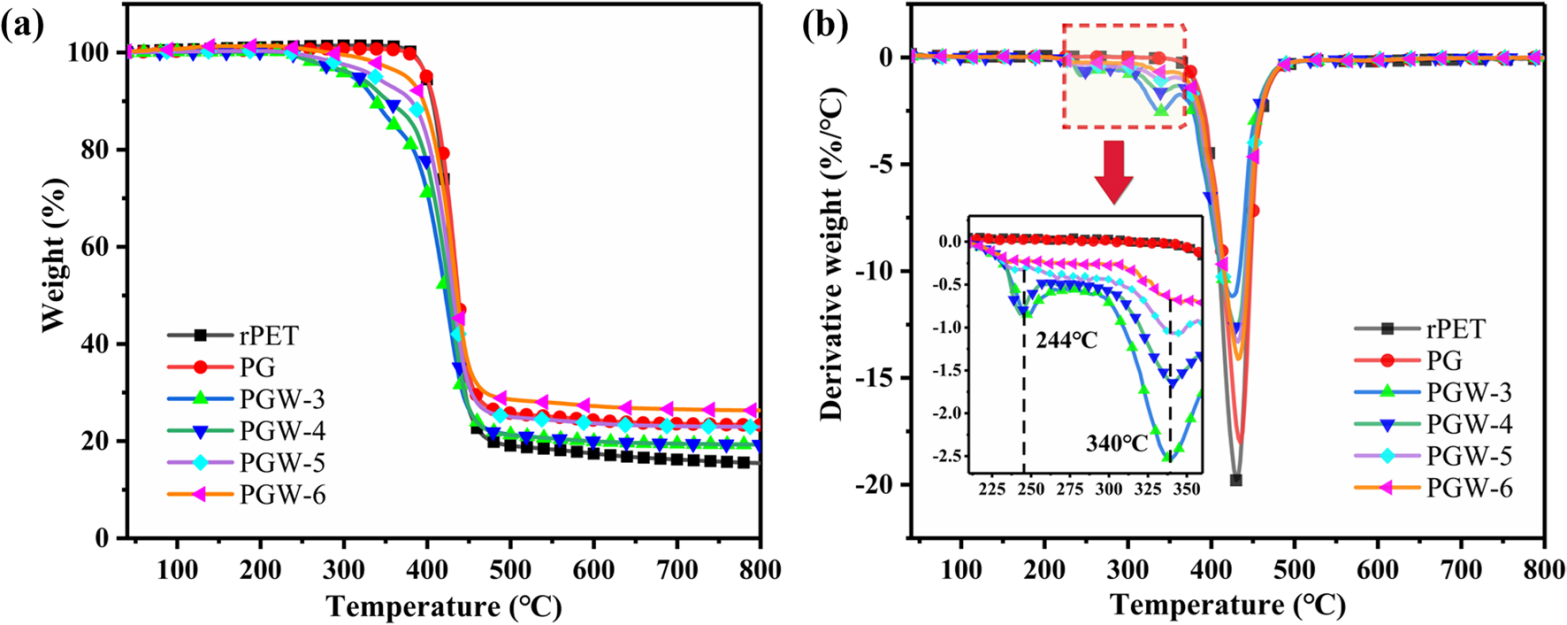
**a** TGA and **b** DTG curves of rPET fabrics, PG and PGW composites

Thermal stability of G/WPU composite fabrics were investigated via TGA (Fig. 5a) and derivative thermogravimetric (DTG) analysis (Fig. 5b). Thermal decomposition process of composite fabric was similar to that of pure rPET fabric. As shown in Fig. 5b, the temperature at maximum degradation rate (*T*_max_) of rPET fabric was recorded as 426 °C, which was attributed to the thermal degradation of alkanes, olefins and aromatics in polyester [47]. After loading graphene nanoplatelets on fabric, *T*_max_ value of PG shifted significantly to 435 °C, mostly due to uniform dispersion of lamellar graphene and its strong adhesion with PET substrate. During thermal degradation, lamellar graphene acted as a physical barrier to prevent the volatilization of small molecule products after degradation [48], thus improving the thermal stability of the composite fabric. After loading G/WPU composites on rPET fabrics, two degradation peaks were occurred at 220–360 °C in DTG curves. With increasing WPU content, the two peak were both enhanced. Actually, DTG peaks at around 244 °C and 340 °C were corresponding to the thermal degradation of hard and soft segments of WPU, respectively [49]. The degradation of rPET in PGW-6 started around 430 °C, slightly higher than that of untreated rPET fabric. The presence of a hydrogen bond between rPET and WPU and the strong interface interaction with PET fabric, was believed to enhance the thermal stability of composite fabric.

### Mechanical performances

**Fig. 6:**
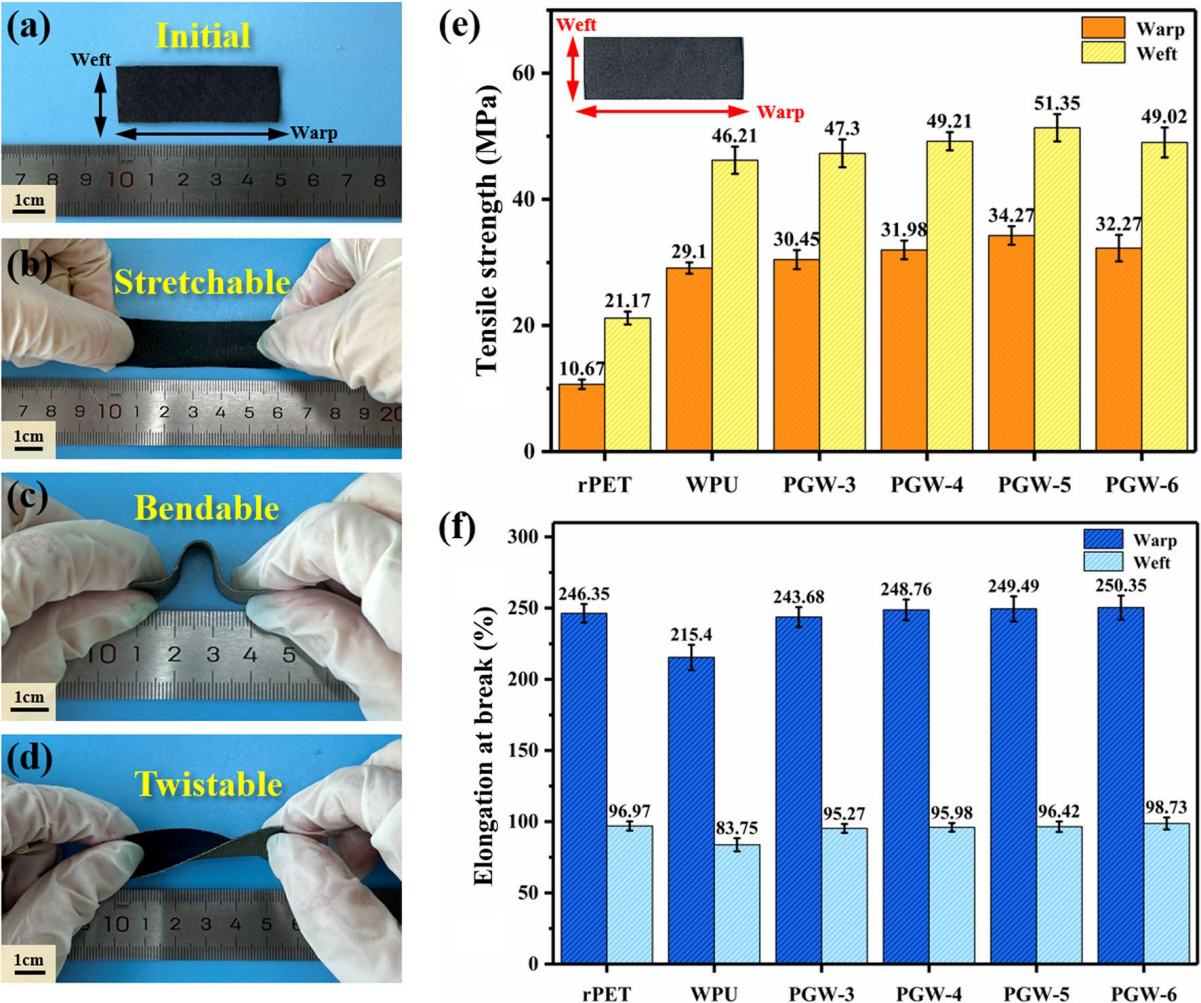
**a**–**d** Images of PGW-5 at different states. Mechanical properties of rPET, WPU and PGW: **e** tensile strength and **f** elongation at break

Mechanical properties of the as-prepared PGW composites were thoroughly investigated. As illustrated in Fig. 6a–Fig. 6d, the obtained PGW-5 fabric exhibited high elasticity and remarkable mechanical toughness, enabling it to undergo stretching, bending, and twisting, which contributing to the application prospects in complex use environments for PGW composite fabrics. Afterwards, the tensile strength and strain of pristine fabric, rPET fabrics coated with graphene (PGW-3, PGW-4, PGW-5 and PGW-6) were quantitatively explored, the corresponding parameters were shown in Fig. 6e, f. With the addition of WPU, both warp and weft breaking strength of the composite fabric were increased from 10.67 MPa to 29.10 MPa and 21.17 MPa to 46.21 MPa respectively mainly due to hydrogen bonds between WPU and rPET. After the addition of graphene, the strength of the fabric was further increased mostly caused by the reinforcement of graphene lamellar structure as a rigid node in in flexible WPU. In Fig. 6e, PGW-5 showed superior mechanical properties, with a warp tensile strength of 34.27 MPa, a greatly 221.2% enhancement compared with rPET. More surprisingly, the elongation at break of the fabric showed limited change (basically close to 250%) after graphene/polyurethane coated, indicating that the composite fabric still maintains its original elasticity. The tensile results revealed that the treatment with G/WPU improved the mechanical properties of rPET fabric. The remarkable mechanical properties, including breaking strength, elongation and flexibility, made PGW suitable for practical applications. Being used as a wearable flexible sensor, these fabrics had a wide strain range and can be used to monitor vital signals such as human joint movement in real time.

### Moisture permeation properties

**Fig. 7:**
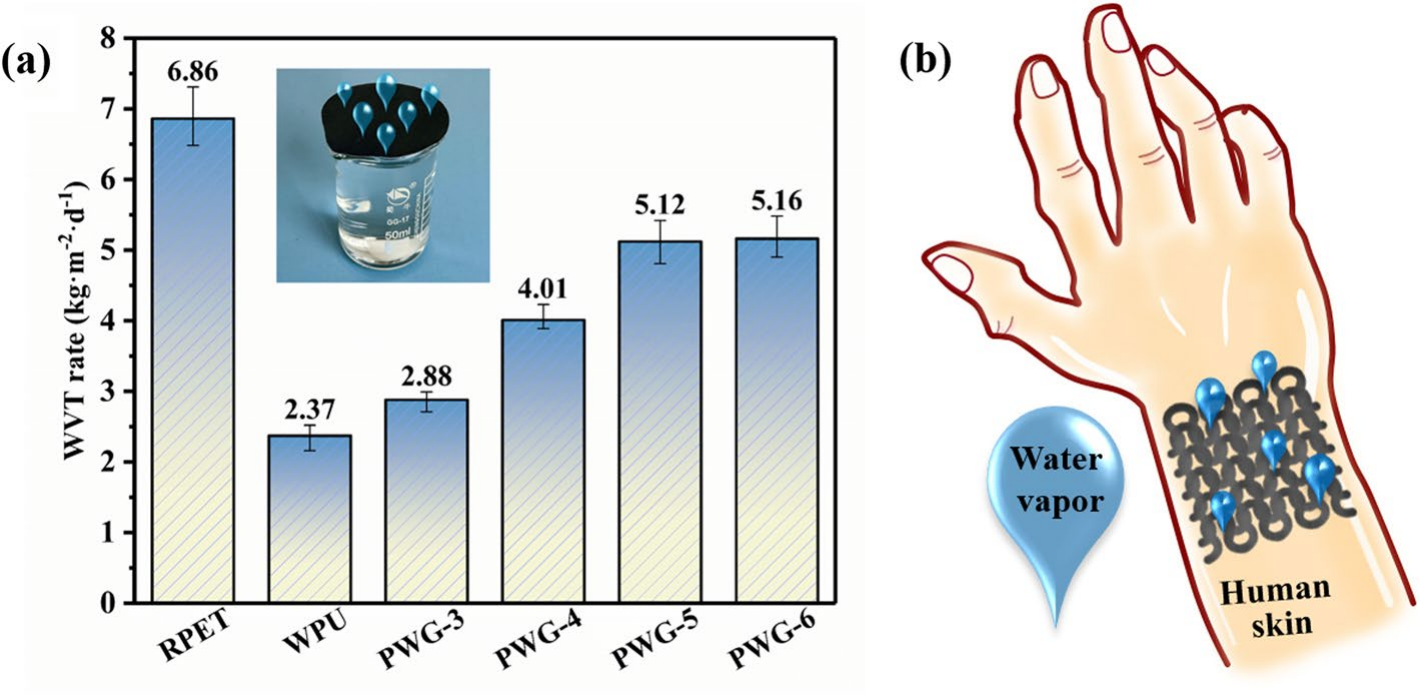
**a** WVT rate of rPET, WPU and PGW, and **b** Schematic of the potential applications of on-skin wearable devices

As seen in Fig. 7a, the original moisture permeability of rPET fabric was recorded as 6.86 due to its porous and loose structure. However, the moisture permeability of original WPU film was really poor. Due to the increase in the content of graphene with hydrophilic groups, the moisture permeability of composited fabrics was gradually improved. In combination with SEM cross-section of Fig. 3f, the flexible composite fabrics preserved three-dimensional porous network structure, which was conducive to the free flow of water vapor. Among them, PGW-5 and PGW-6 flexible composite fabrics possessed higher moisture permeability, with WVT values of 5.12 kg m^−2^ d^−1^ and 5.16 kg m^−2^ d^−1^, respectively. Based on the breathability of the composite fabrics, applications of prolonged wearing comfort and skin surface for signal capture should be a wide range of practical prospects (Fig. 7b).

### Strain sensing behaviors

**Fig. 8:**
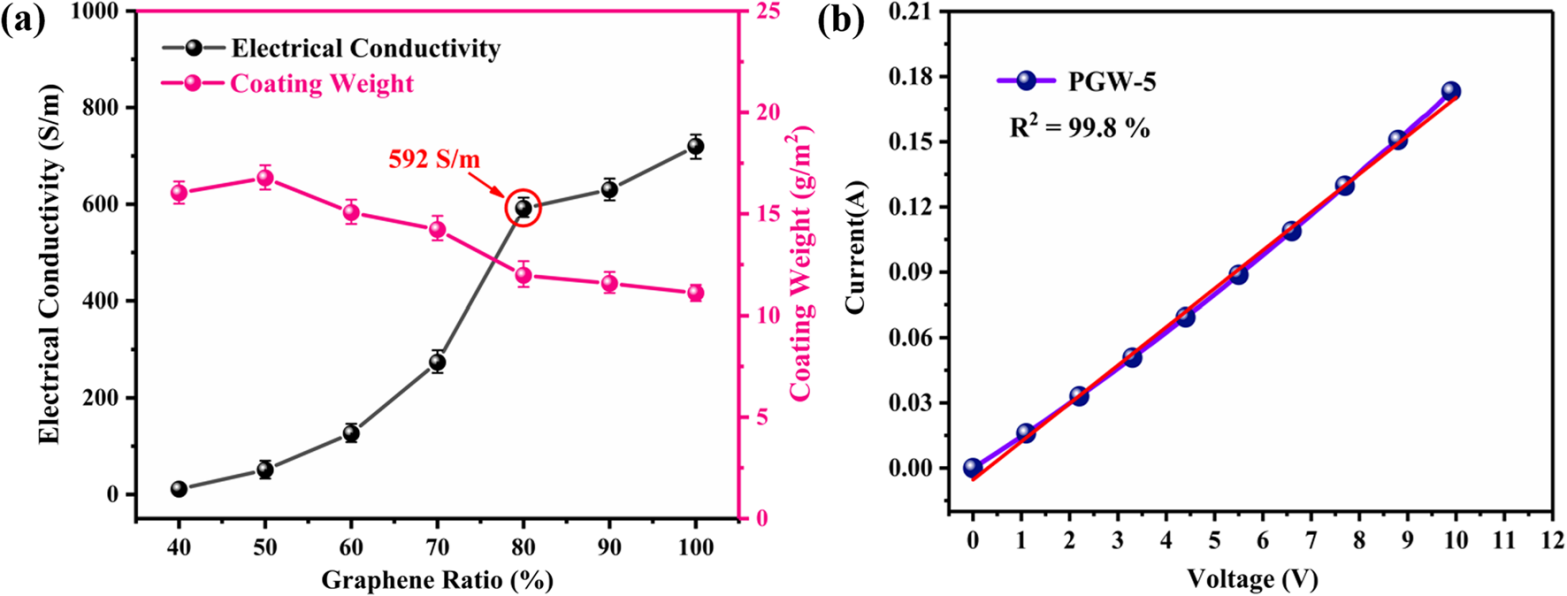
Electromechanical characterizations of PGW fabrics: **a** Conductivity and coating weight and **b** current–voltage characteristics of PGW-5

As a critical factor in electrical conductivity, multiple signal capture applications, and electrothermal conversion performance, electrical conductivity must be taken into account when developing flexible wearable sensors. In Fig. 8a, with increasing graphene proportion in G/WPU, the conductivity of composite fabrics showed an obviously increasing tendency. With G/WPU ratio reaching 8:2, conductivity of the composite fabric had risen to 592 S/m. Furthermore, the trend of increasing conductivity diminished significantly when the graphene content exceeds 80%. While PGW-6 exhibited higher conductivity and moisture permeability compared to PGW-5, its surface coating displayed discontinuities with cracks that were susceptible to peeling off, as shown in Fig. 3e. In addition, in Fig. 8a, the coating weight of composite fabric was slightly decreased with increasing proportion of graphene, maintaining the characteristics of soft and lightweight wearable fabric. After an overall literature research (Additional file 1: Fig. S1), the conductivity and stretchability properties of composite fabric were compared with those of previous researched materials, revealing that PGW-5 exhibited excellent electrical conductivity with 592 S/m and excellent stretchability, which was more beneficial for constructing flexible fabric sensors. The current–voltage (I–V) characteristics of the PGW-5 were presented in Fig. 8b. The current–voltage (I-V) curve of PGW-5 was nearly linear under a specific applied strain, which indicated that the continuous conductive pathways of the PGW-5 was subject to Ohm's law.

Based on excellent mechanical properties, satisfactory moisture permeability and excellent electrical conductivity, adhering to the principle of cost-effectiveness, PGW-5 composite fabric was selected for subsequent sensing performance tests to verify its feasibility as a flexible sensor for multiple signal capture.

**Fig. 9:**
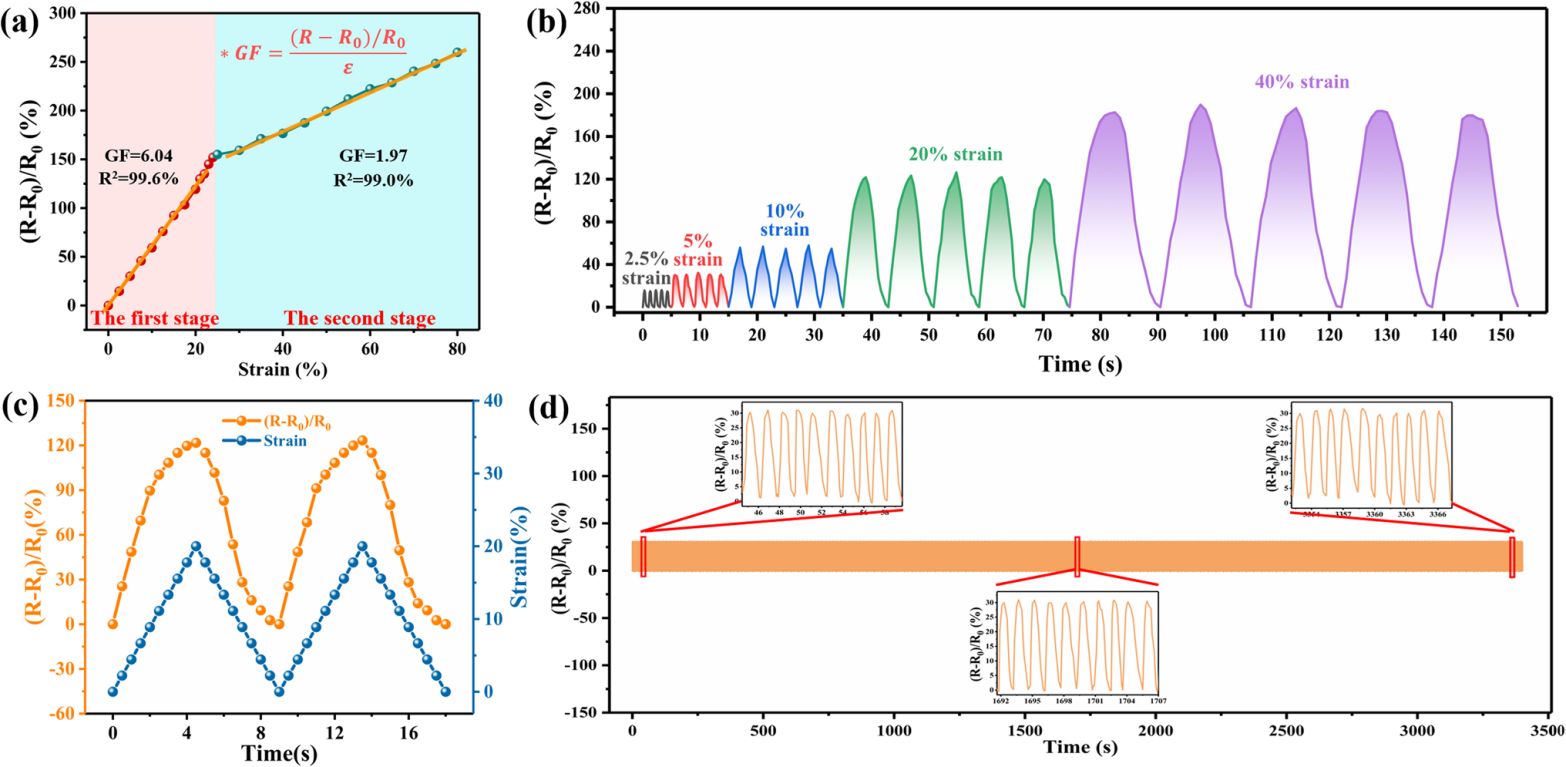
**a** (*R−R*_0_)/*R*_0_ of PGW-5 at various strains, **b** (*R−R*_0_)/*R*_0_ of PGW-5 at 5%/s at various strains **c** (*R−R*_0_)/*R*_0_ and temporal variation of from 0 to 20% with 5%/s, **d** (*R−R*_0_)/*R*_0_ of PGW-5 with 2000 stretching–releasing cycles from 0 to 5% at 10%/s

Moreover, the strain sensing properties of PGW-5 were evaluated in warp direction, and its response was assessed by the rate of electric resistance variation denoted as (*R−R*_0_)/*R*_0_. As shown in Fig. 9a, the (*R−R*_0_)/*R*_0_ of PGW-5 enhanced with the increase of applied strain, and the process of strain response could be classified into two stages. In addition, the strain sensitivity of PGW-5 was employed using gauge factor (GF) as following [50]:

$$GF = \frac{{\left( {R - R_{0} } \right)/R_{0} }}{\varepsilon }$$

where *R* represented the resistance under stretched state, *R*_0_ represented the initial resistance, and *ε* referred to the applied strain. In the initial stage (0–21% strain), (*R−R*_0_)/*R*_0_ increased sharply, and displayed a perfect linear relationship (R^2^ = 99.6%) with strain. This was attributed to that close contact between coils in the unstretched state of PGW-5, and large and disordered number of loops in rPET fabric, resulting in the disorganization of conductive network. After stretching, the coils transfer process changed obviously, the reduction of contact points led to the reduction of the number of conductive circuits formed by the coils, and a rapid increased of resistance change rate (GF = 6.04). In the latter stage (26–80% strain), (*R−R*_0_)/*R*_0_ increased slowly with increasing strain, and (*R−R*_0_)/*R*_0_ with strain exhibited an ideal linear correlation (R^2^ = 99.0%), which was attributed to that with the increase of the strain variable, the contact points between the loops had been separated from each other, and the transfer of coil reached an equilibrium point. The GF in the initial stage was higher than that in the subsequent stage, which manifested those conductive networks were more sensitive to signal capture at smaller strains. The aforementioned results indicated that PGW-5 with high sensitivity and linear correlation with strain, which contributed to being used as flexible sensors. The value of (*R−R*_0_)/*R*_0_ and strain over time, ranging from 0 to 40% with a tensile rate of 5%/s, were depicted in Fig. 9b. (*R−R*_0_)/*R*_0_ underwent an immediate alteration upon the application of strain and reverted back to its initial state during relaxation. The stretching-relaxing process showed a strong correlation between (*R−R*_0_)/*R*_0_ and strain, indicating the excellent responsiveness of PGW-5. As illustrated in Fig. 9c, (*R−R*_0_)/*R*_0_ for PGW-5 under various tensile strains during stretching suggests its synchronization of strain sensing which could enable real-time deformation monitoring. Notably, the maximum value of (*R−R*_0_)/*R*_0_ increased as the strain rose due to the formation of efficient conductive networks within the elastic rPET fabric. To evaluate long-term dynamic stability and durability, PGW-5 underwent testing involving 2000 cycles of stretching and releasing at a rate of 5%/s from 0 to 5%. The consistent signals for (*R−R*_0_)/*R*_0_ in Fig. 9d confirmed that PGW-5 served as a reliable flexible sensor for monitoring strain signals with exceptional dynamic stability and repeatability.

### Omnidirectional motion monitoring

**Fig. 10:**
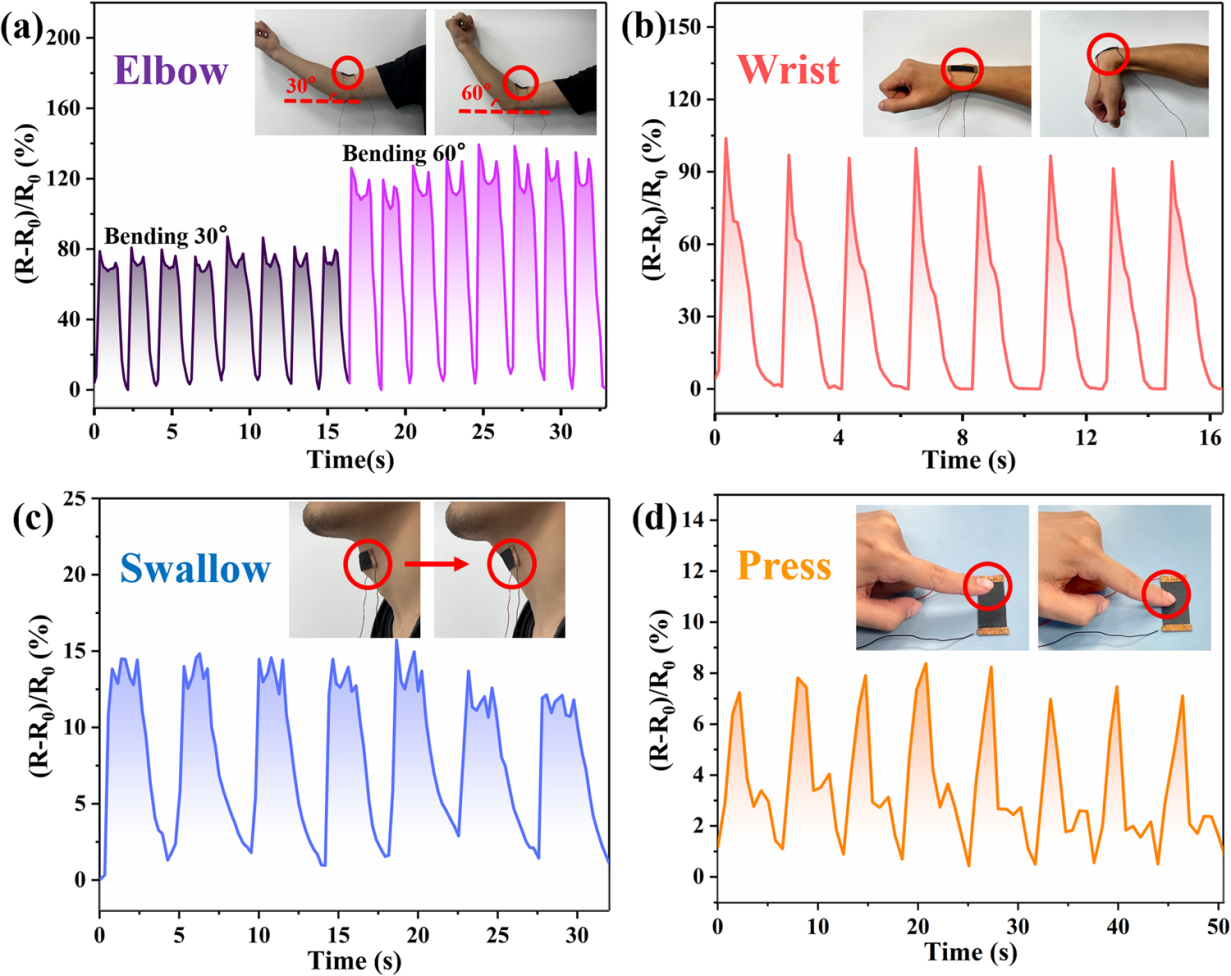
PGW-5 acted as a flexible sensor for real-time monitoring of various physical parts: **a** elbow bending at 30° and 60°, respectively **b** wrist bending **c** throat swallowing, and **d** fingertip pressing

Based on the aforementioned studies, further discussion was conducted regarding the applicability of PGW-5. In Fig. 10a, PGW-5 was affixed to the elbow to assess deformation at different angles (0°, 30°, and 60°). As the elbow flexes, there was an increase in (*R−R*_0_)/*R*_0_, which promptly returned to its original state upon release. Moreover, the maximum of (*R−R*_0_)/*R*_0_ remained consistent at a given bending angle and increases as the bending angle rises, thereby facilitating PGW-5 to monitor diverse degrees of deformations. As illustrated in Fig. 10b, when PGW-5 was attached to human wrist, the resistance change rate (*R−R*_0_)/*R*_0_ increased with increasing wrist bending angle, up to about 114%. During the wrist bending process, the composite fabrics were stretched, and the conductive paths between the internal fibers were converted from mostly parallel to mostly series, resulting in the increase of (*R−R*_0_)/*R*_0_. When PGW-5 was placed at throat area (Fig. 10c), its sensitivity increases during swallowing and quickly recovered to the initial value afterwards. Furthermore, compared to the change in PGW-5 for wrist bending monitoring, (*R−R*_0_)/*R*_0_ for swallowing monitoring was significantly lower at 14.1% with much minor deformation. This proved that PGW-5 as a flexible strain sensor could not only monitor large deformations such as finger bending, but also realized the real-time capture of weak signals such as swallowing action. Moreover, (*R−R*_0_)/*R*_0_ of PGW-5 exhibited different characteristic peaks for different human motion deformation, which could reflect the movement of human body indirectly. The above results proved that it is an ideal material for flexible sensors, which can monitor large deformations such as joints movements as well as subtle deformations. In addition, PGW-5 was an ideal choice for monitoring real-time human movements, capable of effectively monitoring extensive and delicate deformations.

### Electrothermal performance

**Fig. 11:**
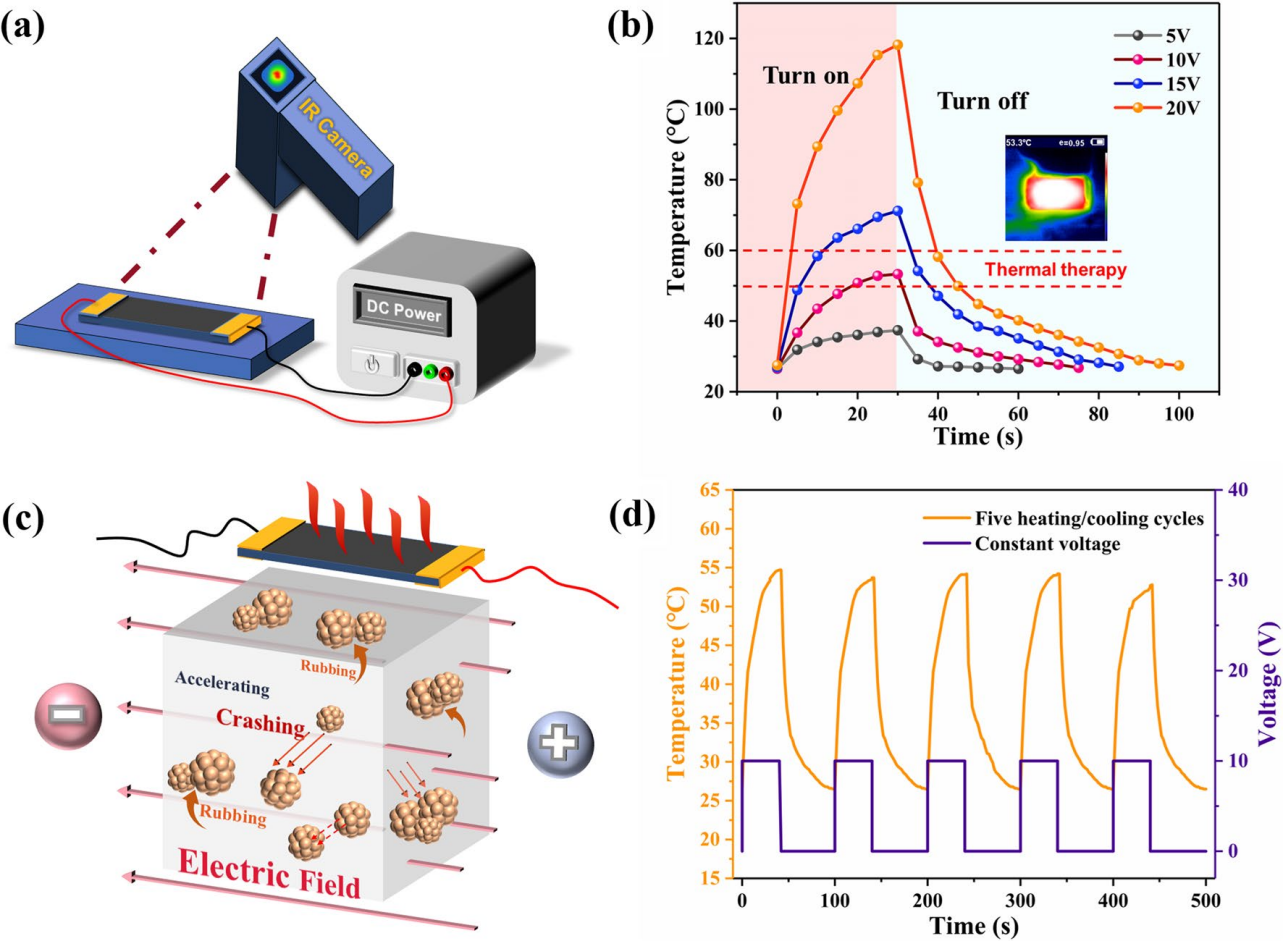
Electrothermal characterization of PGW-5. **a** Diagram of experimental setup for PGW-5 composite heater, **b** time-dependent temperature curves of PGW at different voltages (5, 10, 15 and 20 V), **c** schematic diagram of electrothermal conversion, and **d** Five heating/cooling cycling curves of PGW-5 at an input voltage of 10 V

The Joule heating behavior of PGW-5 was investigated by applying a DC bias power source. The temperature distribution on the surface and the response were observed and recorded by an infrared camera, as shown in Fig. 11a. Time-dependent temperature profiles of PGW-5, as shown in Fig. 11b, revealed that PGW-5 exhibited similar temperature trends under different voltages (5–20 V). It can rapidly generate efficient heat and increase over time in the heating zone. A continuously increasing temperature profile were also observed as the input voltage increased, as illustrated in Fig. 11b. PGW-5 can reach a high temperature of 118.2 °C in 30 s at a voltage of 20 V, demonstrating high heat generation efficiency. Consequently, immediate release of the input voltage led to a rapid reduction of heat, causing the temperature to return to room temperature within a short period of time. As shown in Fig. 11c, when the voltage was applied, the two-dimensional carbon crystal molecules in the structure underwent motion in accordance with “Brownian motion” driven by the micro-current. This motion generated irregular collisions and friction, converting electrical energy into heat energy [51]. Under specific voltage conditions, PGW-5 can generate significant Joule heat due to its relatively low resistance. As a result, the temperature rose rapidly initially and then increased gradually as the power-on time extended. At a voltage of 10 V, PGW-5 generated a temperature of approximately 53.3 °C after 30 s. As known, hot compresses had been proven to enhance and facilitate localized blood circulation, effectively aiding in the treatment of various joint discomforts. However, it was crucial to avoid excessively high temperatures. Typically, the temperature of a hot compress was maintained within the range of 50–60 °C. Accordingly, a voltage of 10 V was selected for the heating and cooling cycle test. Additionally, Fig. 11d illustrated a consistent curve of PGW-5 obtained through five heating/cooling cycles at 10 V. The heater endured repeated heating/cooling, demonstrating a reliable electrothermal stability of PGW-5. From Additional file 1: Fig. S2, it can be observed that the (*R−R*_0_)/*R*_0_ of PGW-5 was minimally affected by temperatures ranging from 30 to 60 °C, indicating that PGW-5 can maintain excellent sensing monitoring during thermotherapy and demonstrate the integration of both thermal therapy and sensing capabilities. Consequently, PGW-5 emerged as a suitable choice for thermotherapy applications such as hyperthermia and thermal underwear.

## Conclusions

In this paper, multi-layer flexible devices were fabricated by utilizing graphene as the conductive component and WPU as the protective layer. The knitted fabrics, which were innovatively prepared through the chemical recycling of waste PET, were employed as substrates. A wearable textile sensor and joule heater device with solid conductive networks was successfully fabricated via the scratch coating method to explore different ratios of graphene and WPU. The PGW-5 functions as a fabric strain sensor, exhibiting exceptional mechanical performance and high conductivity (592 S/m). Surprisingly, PGW-5 demonstrated exceptional sensitivity (GF = 6.04) and remarkable dynamic durability throughout a cyclic stretching-releasing process consisting of 2000 cycles. Furthermore, PGW-5 had the capability to track notable human motions like flexing of the elbow, as well as inconspicuous changes such as swallowing. The technology had significant potential in areas like monitoring human health and wearable electronic skins. Besides, PGW-5 also exhibited a significant electric heating effect. At a voltage of 10 V, it can reach a steady-state temperature of 53.3 °C within 30 s, making it suitable for use as a wearable heater for on-demand thermal therapy.

### Supplementary Information

**Additional file 1: Fig. S1.** Comparison of conductivity with this work: graphite nanoplatelet welded carbon nanotube (GNP-w-CNT), graphene/carboxymethylcellulose-2 (CE/CMC-2), graphene aerogels-2 (GA-2).** Fig. S2.** (*R*−*R*0)/*R*0 of PGW-5 from 0 to 5% at different temperatures (30, 40, 50, 60 °C)

## Data Availability

Data will be made available on request.
