# Supplementary material for: Breathable and wearable graphene/waterborne polyurethane coated regenerated polyethylene terephthalate fabrics for motion sensing and thermal therapy
Source: Discov Nano. 2024 Apr 4;19(1):61. doi: 10.1186/s11671-024-04004-w (PMC10994883; doi:10.1186/s11671-024-04004-w)
Supplement: Supplementary file 1 — Additional file 1: Fig. S1. Comparison of conductivity with this work: graphite nanoplatelet welded carbon nanotube (GNP-w-CNT), graphene/carboxymethylcellulose-2 (CE/CMC-2), graphene aerogels-2 (GA-2). Fig. S2. (R−R0)/R0 of PGW-5 from 0 to 5% at different temperatures (30, 40, 50, 60 °C) [file 11671_2024_4004_MOESM1_ESM.docx]

**Supplementary material**


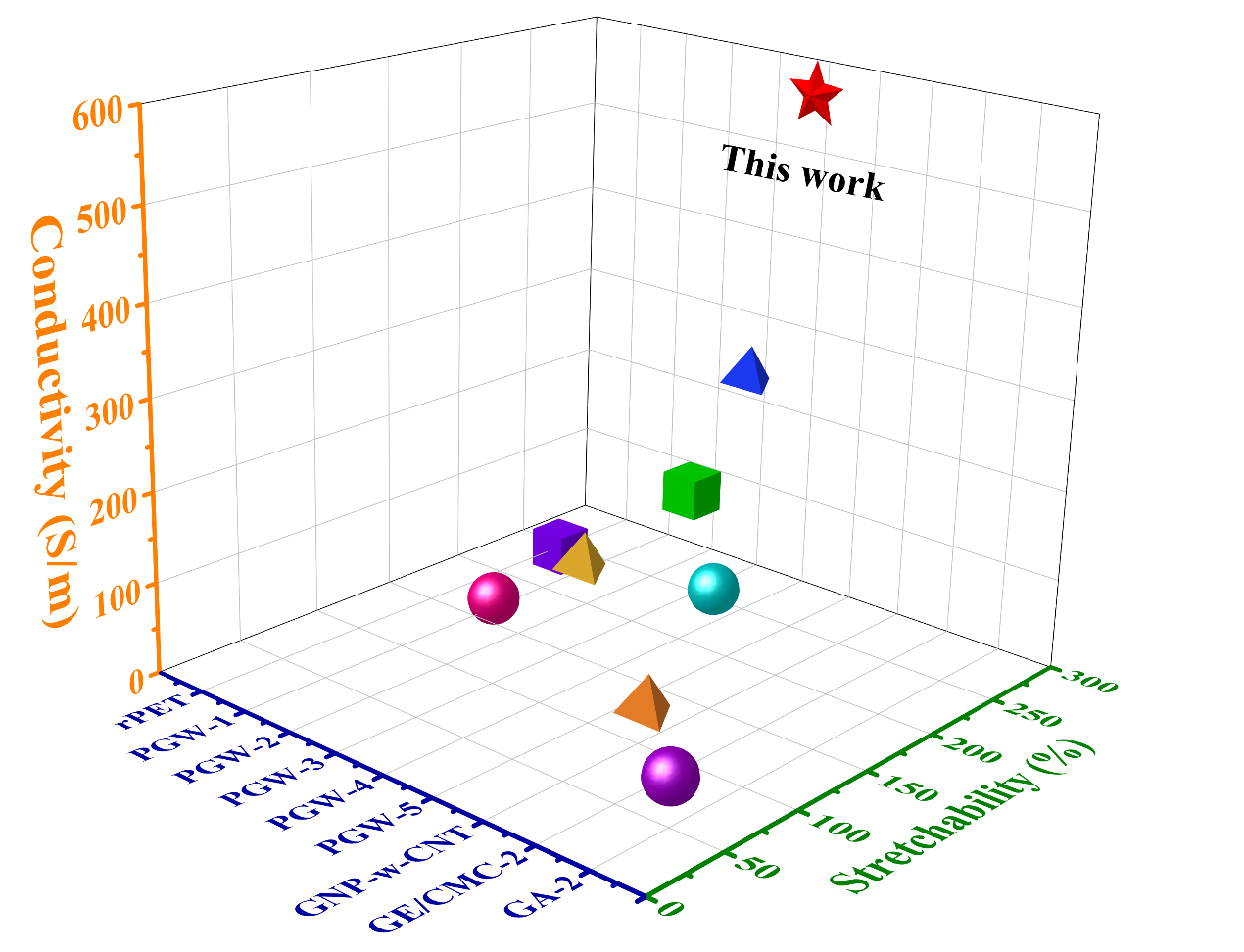


Fig. S1 Overview of conductivity and stretchability with other works: rPET, PGW-1, PGW-2, PGW-3, PGW-4, PGW-5, graphite nanoplatelet welded carbon nanotube (GNP-w-CNT) [1], graphene/carboxymethylcellulose-2 (CE/CMC-2) [2], graphene aerogels-2 (GA-2) [3].

After an overall literature research (Fig. S1), the conductivity and stretchability properties of composite fabric were compared with those of previous researched materials, revealing that PGW-5 exhibited excellent electrical conductivity with 592 S/m, an outstanding property that could allow it to be used as a sensor for signal capture to monitor the full range of human movement or for applications in thermal therapy.

[1] Zhang F, Ren D, Huang L, Zhang Y, Sun Y, Liu D, Zhang Q, Feng W, Zheng Q (2021) Advanced Functional Materials 31: 2107082.<https://doi.org:https://doi.org/10.1002/adfm.202107082>

[2] Huang Z-M, Liu X-Y, Wu W-G, Li Y-Q, Wang H (2017) Journal of Materials Science 52: 12540-12552.<https://doi.org:10.1007/s10853-017-1374-1>

[3] Afroze J D, Abden M J, Yuan Z, Wang C, Wei L, Chen Y, Tong L (2020) Carbon 162: 365-374.<https://doi.org:10.1016/j.carbon.2020.02.057>

**
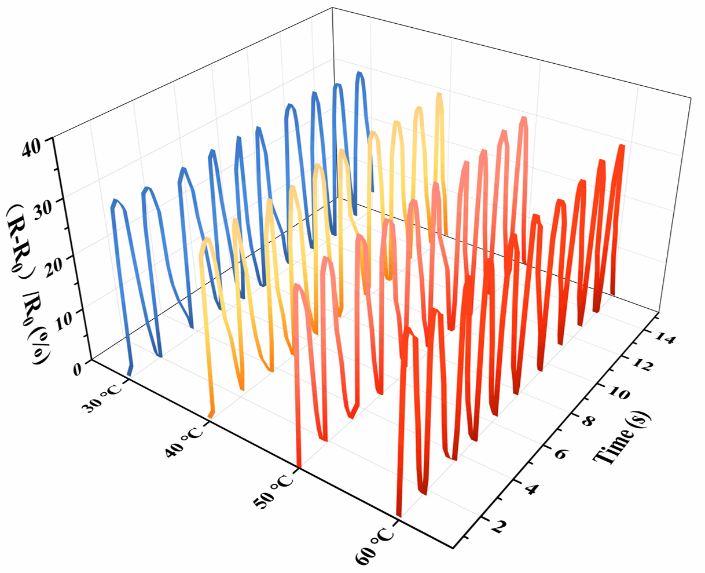
**

Fig. S2 (*R*–*R*_0_)/*R*_0_ of PGW-5 from 0 to 5% at different temperatures (30 °C, 40 °C, 50 °C, 60 °C)

(*R*–*R*_0_)/*R*_0_ of PGW-5 at different temperatures (30 °C, 40 °C, 50 °C, 60 °C), as shown in Fig. S2 above. It had been found that the (*R*–*R*_0_)/*R*_0_ of PGW-5 was minimally affected by temperatures ranging from 30 °C to 60 °C, indicating that PGW-5 can maintain excellent sensing monitoring during thermotherapy and demonstrate the integration of both thermal therapy and sensing capabilities.
